# Visceral Adiposity and Risk of Stroke: A Mendelian Randomization Study

**DOI:** 10.3389/fneur.2022.804851

**Published:** 2022-04-11

**Authors:** Ran Xu, Xinzhi Hu, Tao Wang, Yutong Yang, Nan Jiang, Jichang Luo, Xiao Zhang, Aman B. Patel, Adam A. Dmytriw, Liqun Jiao

**Affiliations:** ^1^Department of Neurosurgery, Xuanwu Hospital, Capital Medical University, Beijing, China; ^2^China International Neuroscience Institute (China-INI), Beijing, China; ^3^Department of Human Anatomy, Histology and Embryology, Peking Union Medical College Hospital, Peking Union Medical College, Institute of Basic Medical Sciences, Chinese Academy of Medical Science, Beijing, China; ^4^Faculty of Medicine, Imperial College London, National Heart and Lung Institute, London, United Kingdom; ^5^Neuroendovascular Program, Massachusetts General Hospital, Boston, MA, United States; ^6^Neuroradiology and Neurointervention Service, Brigham and Women's Hospital Harvard Medical School, Boston, MA, United States; ^7^Department of Interventional Neuroradiology, Xuanwu Hospital, Capital Medical University, Beijing, China

**Keywords:** visceral adipose tissue (VAT), ischemic stroke, intracranial hemorrhage (ICH), mendelian randomization (MR), single nucleotide polymorphism (SNP)

## Abstract

**Purpose:**

In recent years, metabolic syndrome has risen in prevalence and brought a heavy disease burden to modern society. As the representative aspect of metabolic syndrome, obesity has been shown to be related to an increased risk of stroke. Given that visceral adipose tissue (VAT) forms the fundamental basis of central obesity, we sought to explore a causal relationship between VAT and stroke by using mendelian randomization (MR) methods.

**Methods:**

Based on two large genome-wide association studies (GWAS) including 325,153 and 35,762 cases of VAT and stroke, respectively, we conducted a MR study which has the inherent advantage of reducing the noise of confounding and reverse causation.

**Results:**

VAT had a significant causal association with ischemic stroke (OR, per 1kg increase in VAT mass, 1.30; 95% CI, 1.18 ~ 1.45; *P* = 5.87E-07) as opposed to intracranial hemorrhage (ICH) (OR, 1.15; 95% CI, 0.70 ~ 1.88, *P* = 5.81E-01) as evaluated with inverse-variance weighting (IVW). Regarding subtypes of ischemic stroke, there was a significant causal effect for cardioembolic stroke (OR, 1.34; 95% CI, 1.13 ~ 1.58, *P* = 8.07E-04), and potential causal effect for small-vessel stroke (OR, 1.32; 95% CI, 1.06 ~ 1.65, *P* = 1.39E-02) and large-artery atherosclerotic stroke (OR, 1.33; 95% CI, 1.03 ~ 1.70, *P* = 2.59E-02).

**Conclusions:**

This study provides potential evidence for a causal role of VAT in ischemic stroke and could suggest novel genetical therapeutic strategies for distinct subtypes of ischemic stroke.

## Introduction

Stroke is the second leading cause of death and long-term disability (1). Despite the remarkable advances in medical and intervention treatment, the prognosis of ICH and ischemic stroke may still at many times be dismal. There is a strong association between obesity and hypertension, diabetes mellitus, as well as high density lipoprotein (HDL) cholesterol. Thus, obesity has been considered a well-recognized risk factor for stroke (2), and would be a promising target for the prevention of stroke. However, the underlying mechanisms of obesity-related stroke remain to be investigated.

From an etiological point of view, metabolic syndrome is considered a major driver of obesity. Its pathological basis related to levels of insulin resistance, which was found to be mainly correlated with VAT rather than BMI, weight or degree of subcutaneous fat (3). VAT is the accumulation of adipose tissue on internal organs and is frequently present in patients with fatty liver disease, pancreatitis and stroke. VAT is considered an abnormal type of obesity which is most prevalent in the setting of metabolic syndrome. Increased VAT has been demonstrated to increase the risk of stroke (4), and it has been shown that lower VAT is related with more favorable clinical outcome in patients with acute stroke treated with intravenous thrombolysis (5). Therefore, identification of a causal relationship between VAT and stroke can shed light on the underlying pathogenic mechanism of stroke. However, its causality with stroke has not yet been established.

Mendelian randomization (MR) analysis is emerging as an ideal approach to determine causality between exposures and outcomes using genetic variants as the instrumental variable (6). Thus, we sought to investigate the causality between VAT and stroke with MR analysis.

## Methods

### Study Design

A two-sample MR analysis was performed to investigate the causal relationship between VAT and stroke. All of the included studies received approval from the original institutional review board and ethics committees. For this analysis, only summarized data from the consortium were extracted.

Mendelian randomization uses genetic variants as instrumental variables to estimate the causal effect of exposure on an outcome. A genetic variant is a valid instrumental variable if it is associated with the risk factor of interest and unrelated with any confounder of the exposure-outcome association. Besides, the variant is supposed to be conditionally independent of the outcome given the risk factor and confounders. To avoid violating the instrumental variable assumptions, Mendelian randomization analysis is generally based on genetic variations associated with single exposure. In practice, however, many variants are pleiotropic, that is, associated with multiple risk factors. To include information on polymorphic variants and provide a more robust analysis without reducing validity, we employ the multiple mendelian randomization analysis to distinguish the effect of VAT from waist-hip-ratio (WHR) and BMI. Specifically, for each variant in multiple mendelian randomization, we assume that the variant is associated with 1 or more of the risk factors and not related to a confounder of any of the risk factor–outcome associations. Also, the variant is supposed to be conditionally independent of the outcomes.

### Genetic Instrument Selection

SNPs associated with VAT at genome-wide significance (*P* < 5 × 10^−8^) from a large-scale GWAS comprising 325,153 individuals of European ancestry concerning VAT served as instrumental variables (7). This large-scale GWAS employed nonlinear prediction models from related variables which are more easily to be evaluated than VAT itself. The models were calibrated through a large training dataset, with VAT measured by DXA in 4,198 individuals of white British ancestry from the UK Biobank (UKBB). All SNPs were clumped with a 10,000 kB window to a threshold of *R*^2^ <0.1 to ascertain independence between genetic variants. Pleiotropy of the SNPs was checked in the PhenoScanner database (July 9th, 2020) (8). SNPs associated with the following possible risk factors for ischemic stroke were pruned: systolic and diastolic blood pressure, high-density lipoprotein cholesterol, type 2 diabetes mellitus, and smoking. The strength of the instrument was represented by the F-statistic, which was calculated based on the proportion of phenotypic variance (R^2^) explained by each SNP. The SNPs used in the multivariable mendelian randomization satisfy the following standards: significantly associated with BMI and WHR in the Genetic Investigation of ANthropometric Traits (GIANT) dataset, being included as the instruments for VAT in the univariable mendelian randomization. SNPs were excluded in the following situation, palindromic, not available in the MEGASTROKE consortium, no suitable proxy (with linkage disequilibrium *r*^2^ > 0.9) as alternative. The characteristics of adopted SNPs were listed in Supplementary Table 1.

### Outcome Data Sources

Genetic association estimates for ischemic stroke were obtained from the MEGASTROKE Consortium (9) including 34,217 cases of ischemic stroke and 406,111 controls of European ancestry. According to the acute stroke treatment (TOAST) criteria, ischemic stroke was further subdivided into large-artery atherosclerotic ischemic stroke (*n* = 4,373), cardioembolic ischemic stroke (*n* = 7,193), and small-vessel ischemic stroke (*n* = 5,386) (10). For ICH, we extracted summary statistics from the International Stroke Genetics Consortium (ISGC) meta-analysis including 1,545 cases and 1,481 controls of European ancestry (11).

### Statistical Analysis

For individual SNPs, MR estimates were determined using the ratio of coefficients method with the standard error calculated with the delta method. The primary analyses were conducted by summarizing the ratio estimates under a random-effects model inverse-variance weighting (IVW) models, This method assumed that there are no horizontal pleiotropic effects of the genetic instruments on the outcome. Sensitivity analyses fixed-effects IVW, MR-Egger regression and penalized weighted median methods.

Horizontal pleiotropic effects were estimated by MR-Egger intercept test. MR-Egger estimates represent the pleiotropy-adjusted MR estimate (12). An intercept value for MR-Egger regression that deviates significantly from zero (*P* < 0.05) was used to indicate horizontal pleiotropy. The Bowden I^2^ statistic was used to assess the validity of the instruments and measure the instrument strength for the MR-Egger regression method. An I^2^ value below 50% was used to indicate MR-Egger regression tendency to underestimate causal effect. We also performed the penalized weighted median estimates, which provides additional robustness by penalizing the weights of candidate instruments with heterogeneous ratio estimates in the weighted regression model. Funnel plots were also applied to visualize heterogeneity, with asymmetry about the vertical line indicating possible directional pleiotropy. Finally, we applied the MR pleiotropy residual sum and outlier (MR-PRESSO) method to confirm potential outliers.

To examine whether the sample size is sufficient to perform the MR analyses, we conducted power calculations with a published and validated calculator (13). We employed a multivariable MR model using WHR and BMI as a covariate based on the same instruments since there was an overlap between VAT mass-associated loci and loci associated with other adiposity phenotypes. Genetic data concerning inverse standard normal transformed BMI and inverse standard normal transformed WHR of European ancestry were obtained from the GIANT. The instruments for multivariable mendelian randomization analysis were generated by first filtering the hundreds of SNPs associated with BMI or WHR for those present across the VAT datasets. The instruments also satisfy a similar set of assumptions of univariable mendelian randomization, but in this case the variants must be exclusively associated not with a single risk factor but with three measured risk factors (14).

All results were transformed by exponentiation for representation as ORs, and 95% CIs for stroke per 1kg increase in VAT mass. We adopted a Bonferroni-corrected significance threshold of α = 0.05/5 to adjust for multiple testing. Per convention, *p*-values between 0.05 and 0.01 suggest potential causal significance between exposure and outcome. All statistical analyses were accomplished on R version 4.0.0 with R packages Mendelian Randomization, Two Sample MR and MR-PRESSO.

### Standard Protocol Approvals, Registrations, and Patient Consent

This study included summarized data published by multiple GWAS. Thus, consent was derived via the corresponding studies. All reports in our study were following the STROBE (Strengthening the Reporting of Observational Studies in Epidemiology) guideline for the reporting of MR studies.

### Data Availability Statement

All data used for the analyses are available through the original studies. The analysis codes used in this study are available on request to corresponding authors.

## Results

156 SNPs and 129 SNPs were used as instruments for the analysis for ischemic stroke and ICH. All of the instruments are associated with VAT and independent of the most confounders of VAT accept BMI and WHR. All of the instruments are independent of the outcomes. In addition to instrumental variable assumptions, there are additional assumptions and considerations. These include assumptions of homogeneity, which is crucial to obtain causal effect point estimates. As the value I^2^ statistic were all below 40% (Table 1), indicating moderate heterogeneity, we assume it justified the similarity of the genetic variant–exposure associations between the exposure and outcome samples.

**Table 1:** Casual relationships between VAT and stroke.

**Outcome**	**Sample size (cases/controls)**	**SNP (n)**	**OR (95%)**	***P*-value**	**Intercept P**	**I^**2**^ (%)**
**AIS**	440,328 (34,217/406,111)					
IVW (fixed effects)		156	1.30 (1.20~1.42)	1.71E-09	NA	32
IVW		156	1.30 (1.18~1.45)	5.87E-07	NA	32
MR Egger		156	1.19 (0.81~1.75)	3.68E-01	6.38E-01	32
PWM		156	1.18 (1.03~1.35)	1.68E-02	NA	NA
MR PRESSO		156	1.30 (1.18~1.45)	1.57E-06	NA	NA
**CES**	413,304 (7,193/40,6111)					
IVW (fixed effects)		156	1.34 (1.13~1.58)	6.19E-04	NA	5
IVW		156	1.34 (1.13~1.58)	8.07E-04	NA	5
MR Egger		156	1.24 (0.65~2.34)	5.15E-01	8.05E-01	5
PWM		156	1.30 (1.01~1.67)	4.23E-02	NA	NA
MR PRESSO		156	1.34 (1.13~1.58)	1.01E-03	NA	NA
**LAS**	410,484 (4,373/406,111)					
IVW (fixed effects)		156	1.33 (1.07~1.65)	9.48E-03	NA	27
IVW		156	1.33 (1.03~1.70)	2.59E-02	NA	27
MR Egger		156	1.51 (0.59~3.86)	3.93E-01	7.84E-01	27
PWM		156	1.43 (1.03~1.99)	3.26E-02	NA	NA
MR PRESSO		156	1.33 (1.03~1.70)	2.74E-02	NA	NA
**SVS**	411,497 (5,386/411,497)					
IVW (fixed effects)		156	1.32 (1.08~1.61)	6.35E-03	NA	19
IVW		156	1.32 (1.06~1.65)	1.39E-02	NA	19
MR Egger		156	1.46 (0.64~3.33)	3.73E-01	8.08E-01	20
PWM		156	1.12 (0.81~1.55)	4.86E-01	NA	NA
MR PRESSO		156	1.32 (1.06~1.65)	1.50E-02	NA	NA
**ICH**	3,026(1,545/1,481)					
IVW (fixed effects)		129	1.15 (0.71~1.86)	5.71E-01	NA	6
IVW		129	1.15 (0.70~1.88)	5.81E-01	NA	6
MR Egger		129	2.28 (0.34~15.11)	3.94E-01	4.62E-01	6.
PWM		129	1.32 (0.66~2.66)	4.31E-01	NA	NA
MR PRESSO		129	1.15 (0.70~1.88)	5.82E-01	NA	NA

*IVW, inverse variance weighted; PWM, penalized weighted median; OR, odds ratio; AIS, all ischemic stroke; CES, cardioembolic stroke; LAS, large artery stroke; SVS, small vessel stroke; ICH, intracranial hemorrhage*.

MR causal effect estimates for per 1 kg increase in VAT mass and the risk of stroke are shown in Table 1. In summary, we identified that genetically higher VAT was strongly associated with higher risk of ischemic stroke according to the analysis by IVW method (OR, per 1 kg increase in VAT mass, 1.30; 95% CI, 1.18~1.45; *P* = 5.87E-07). However, no evidence for causal relationship was found between VAT mass and ICH (OR, 1.15; 95% CI, 0.70~1.88, *P* = 5.81E-01).

According to established concept, ischemic stroke is a polyetiological disease with remarkable differences between subtypes regarding both risk factors as well as outcome (15). Thus, we also explored the causal relationship between VAT and subtypes of ischemic stroke which were categorized into cardioembolic ischemic stroke, small-vessel ischemic stroke and large-artery atherosclerotic ischemic stroke. Among three subtypes of ischemic stroke, a significant causal effect of VAT on the risk of cardioembolic ischemic stroke (OR, 1.34; 95% CI, 1.13~1.58, *P* = 8.07E-04, using the IVW method), as well as a potential causal effect on small-vessel ischemic stroke (OR, 1.32; 95% CI, 1.06~1.65, *P* = 1.39E-02) and large-artery atherosclerotic ischemic stroke (OR, 1.33; 95% CI, 1.03~1.70, *P* = 2.59E-02) were identified after correction for multiple testing. Power calculation revealed a 100% statistical power to detect an OR of 0.8 (or 1.2). Multivariable MR analyses indicated that VAT mass is causally correlated to the risk of ischemic stroke (OR, 1.31, 95%CI 1.18~1.46, *P* < 0.001) and independent of BMI and WHR. As for the subtypes, we found significant causal effect of VAT on CES (OR 1.33, 95% CI 1.12~1.58, *P* = 0.001) and potential effect on LAS (OR 1.36, 95% CI 1.06~1.73, *P* = 0.015) (Table 2), which is consistent with the univariable mendelian randomization result.

**Table 2:** Results of Multivariable mendelian randomization using IVW method.

**Outcome**	**SNP(n)**	**OR(95%)**	***P*-value**
**AIS**			
VAT/adjusted BMI	153	1.04 (0.74~1.45)	8.25E-01
VAT/adjusted WHR	153	1.02 (0.60~1.75)	9.33E-01
VAT	153	1.31 (1.18~1.46)	<0.001
**CES**			
VAT/adjusted BMI	153	1.07 (0.62~1.84)	8.20E-01
VAT/adjusted WHR	153	1.09 (0.45~2.64)	8.57E-01
VAT	153	1.33 (1.12~1.58)	1.00E-03
**LAS**			
VAT/adjusted BMI	153	1.41 (0.64 ~ 3.10)	3.91E-01
VAT/adjusted WHR	153	0.40 (0.11 ~ 1.42)	1.56E-01
VAT	153	1.36 (1.06 ~ 1.73)	1.50E-02
**SVS**			
VAT/adjusted BMI	153	1.63 (0.80 ~ 3.31)	6.35E-03
VAT/adjusted WHR	153	0.46 (0.15 ~ 1.46)	1.39E-02
VAT	153	1.34 (1.08 ~ 1.67)	3.73E-01
**ICH**			
VAT/adjusted BMI	126	0.24 (0.04 ~ 1.57)	1.36E-01
VAT/adjusted WHR	126	9.64 (0.49 ~ 190.4)	1.36E-01
VAT	126	1.16 (0.69 ~ 1.93)	5.79E-01

The MR estimates with fixed effects IVW exhibited similar results in terms of the relationship between increase of VAT mass and the risk of stroke. The results from penalized weighted median methods reached the similar conclusion (Table 1). In addition, the intercept of the MR-Egger regression suggested no evidence of horizontal pleiotropy (*P* > 0.05). As the value I^2^ statistic were all below 50%, it is reasonable to have more confidence in the result calculated by IVW methods.

**Figure 1:**
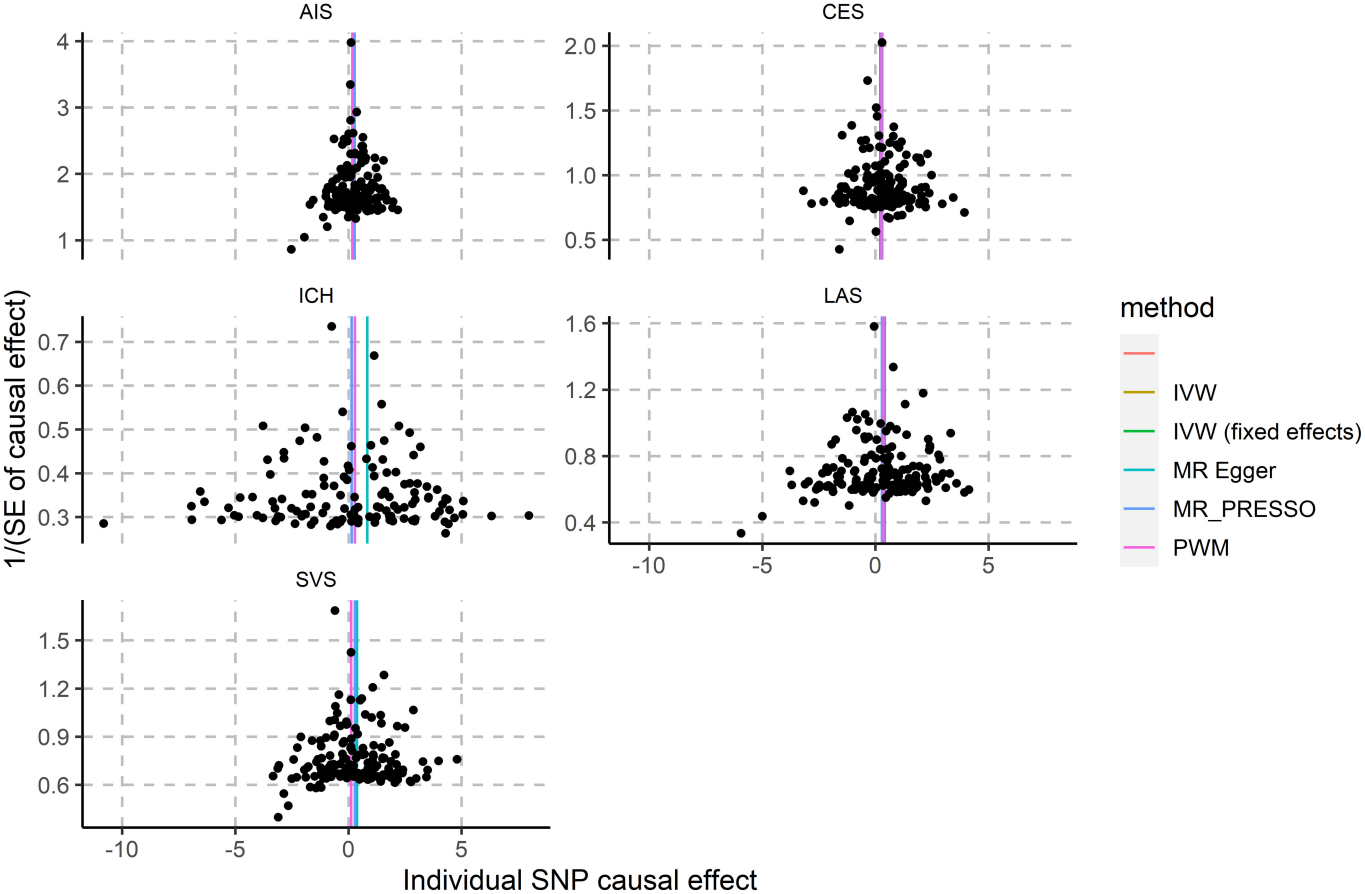
Association of genetically-predicted VAT mass with ICH and ischemic stroke subtypes. AIS, all ischemic stroke; CES, cardioembolic stroke; LAS, large artery stroke; SVS, small vessel stroke.

We found no outlier in the funnel plot (Figure 1), which is also supported by the MR-PRESSO outlier test. An apparent effect of VAT on the risk of ischemic stroke were identified in MR-PRESSO (OR 1.33; 95% CI 1.20~1.47; *P* = 1.57E-06), particularly the subtype of cardioembolic ischemic stroke (OR 1.32; 95% CI 1.12~1.55; *P* = 1.01E-03).

## Discussion

By leveraging large-scale genetic association data, we demonstrated VAT had a significant causal association with ischemic stroke (and not ICH) with multiple MR approaches. For ischemic subtypes divided by TOAST criteria, VAT had a significant causal association with cardioembolic ischemic stroke which is the most frequent etiology of stroke. While the causal association between VAT and small-vessel ischemic stroke as well as large-artery atherosclerotic ischemic stroke was potential. The topic focused on causal association between VAT and stroke was investigated by Yan et al. (16) by extracting the data from MEGASTROKE Consortium. However, we included data from both MEGASTROKE Consortium and International Stroke Genetics Consortium (ISGC) meta-analysis. Therefore, we identified extra novel findings beyond the scope of Yan's work. In detail, we divided stroke into two main subtypes including ICH and ischemic stroke, while ICH was not included in Yan B's study. Thus, we clearly concluded that VAT has a causal relationship with ischemic stroke rather than ICH, which could provide a practicable reference for preventing the incidence of ischemic stroke.

There are distinct populations which are more likely to be affected by different subtypes of ischemic stroke. For example, cardioembolic ischemic strokes tend to occur in older individuals with a history of atrial fibrillation, while large-artery atherosclerotic ischemic stroke was common to detect in middle-aged individuals. In addition, the occurrence of subtypes of ischemic stroke is affected by ethnic and geographical variations. In a previous study performed in Arab and South Asian patients, notable differences between these patients were observed with respect to the number of risk factors for stroke (17). The influence of ethnicity on the occurrence of ischemic subtypes must be considered.

The association between obesity and cardioembolic ischemic stroke has been clarified in the past decade (18). Cardioembolic ischemic strokes were significantly positively associated with degree of obesity as measured by mean BMI or WHR, although the specific underlying mechanism remains unclear (18). According to a previous investigation based on 934 participants in the Atherosclerosis Risk in Communities (ARIC) study, central obesity and the degree of insulin resistance are associated with incident lacunar disease (19). In fact, a case-control study performed in North Manhattan over a decade prior to that claimed that abdominal obesity could serve an independent, potent risk factor for ischemic stroke across all race-ethnic groups. Moreover, abdominal obesity is a stronger risk factor than BMI and has a greater effect among younger persons (20). Our findings are consistent with these previous epidemiological studies and further consolidate the influence of obesity on the incidence of ischemic stroke.

According to a previous follow-up study in a Chinese population (21), abdominal obesity had the highest HR (2.12, *P* < 0.001) for ischemic stroke, followed by metabolic syndrome (HR 1.65, *P* < 0.001). However, people with hypertension had the highest HR (2.17, *P* < 0.001) for ICH, followed by abdominal obesity (HR 1.83, *P* < 0.001). Another study performed in neurologically healthy people in Korea, higher VAT/subcutaneous adipose tissue (SAT) ratios were found to be independent predictors of cerebral microbleeds, a potential precursor of ICH (22). We noticed that the above two studies were performed in Asians, while our findings were achieved by analyzing genetic data of Europeans. This may be a potential explanation that no causal associations were found between VAT and ICH.

BMI has been widely used to measure the severity of obesity. A prospective cohort study indicated that BMI of 30 or greater had a higher relative risk of ischemic stroke of 1.95 (95% CI, 1.39–2.72) when compared with participants who had a BMI less than 23 (23). However, it remains controversial whether BMI is causally linked to ischemic stroke or its subtypes. Notably, fat mass index and fat-free mass index was positively and inversely associated with the incidence of ischemic stroke, respectively, which also suggests a critical role of adiposity distribution in the development of ischemic stroke. Our study further consolidates the critical role of VAT on the incidence of ischemic stroke, which ideally paves the way for future mechanistic studies on VAT.

There are several limitations inherent to the present study. Firstly, this MR study was based on linear effect assumptions which were decided upon by the study design of the original GWAS utilized. Thus, we cannot investigate whether VAT and ischemic stroke has a nonlinear relationship with MR. In addition, the different extent of causal association between VAT with subtypes of ischemic stroke may attribute to the more limited sample sizes for various subtypes in the initial GWAS discovery. Secondly, the cases and controls in this study were of European ancestry, and thus despite previous suggesting VAT results can be extended to other populations (e.g., Asian), we cannot corroborate that with certainly in this study (24–26). Future studies should enroll diverse participants as possible. Additionally, it is not possible to identify gender-specific influences of VAT on ischemic stroke since the individual patient data from the GWAS datasets are not available.

In summary, we clarify the causal relationship between VAT and stroke. In particular, our findings are of direct clinical significance in the guidance of risk factor control of ischemic stroke. Future studies for underlying molecular mechanisms between VAT and ischemic stroke are needed.

## Data Availability Statement

The original contributions presented in the study are included in the article/Supplementary Material, further inquiries can be directed to the corresponding authors.

## Author Contributions

RX, AD, and LJ: conception and design. XH and TW: development of methodology. JL and XZ: acquisition of data. AP, NJ, and YY: analysis and interpretation of data. RX and XH: writing of the manuscript. LJ, AP, and AD: study supervision. All authors contributed to the article and approved the submitted version.

## Funding

This work was supported by the Ministry of Science and Technology of the People's Republic of China (2016YFC1301700), Capital Medical University Science Program for Fostering Young Scholars (1210020222), and Xuanwu Hospital Science Program for Fostering Young Scholars (QNPY2020010).

## Conflict of Interest

The authors declare that the research was conducted in the absence of any commercial or financial relationships that could be construed as a potential conflict of interest.

## Publisher's Note

All claims expressed in this article are solely those of the authors and do not necessarily represent those of their affiliated organizations, or those of the publisher, the editors and the reviewers. Any product that may be evaluated in this article, or claim that may be made by its manufacturer, is not guaranteed or endorsed by the publisher.

## Abbreviations

VAT: visceral adipose tissue
ICH: intracranial hemorrhage
BMI: body mass index
MR: mendelian randomization
SNP: single nucleotide polymorphism
WHR: waist-hip-ratio
OR: odds ratio.
