# Supplementary material for: Visceral Adiposity and Risk of Stroke: A Mendelian Randomization Study
Source: Front Neurol. 2022 Apr 11;13:804851. doi: 10.3389/fneur.2022.804851 (PMC9035635; doi:10.3389/fneur.2022.804851)
Supplement: Supplementary file 1 [file Table_1.DOCX]

**Supplementary Table 1. Genetic variants used as instrumental variables for VAT**

| snp | chr | pos | a1 | a2 | beta | se | P | F Statistics | proxy SNP(R^2^) |
| --- | --- | --- | --- | --- | --- | --- | --- | --- | --- |
| rs10182458 | 2 | 25150641 | G | A | 0.02614085 | 0.002481117 | 5.9003E-26 | 111.0049383 |  |
| rs10187101 | 2 | 50742227 | T | C | -0.017220821 | 0.002584759 | 2.69305E-11 | 44.38792919 |  |
| rs10756714 | 9 | 15885041 | G | A | -0.019681361 | 0.002498507 | 3.3467E-15 | 62.0506567 |  |
| rs10773302 | 12 | 123043145 | G | T | -0.017215152 | 0.002798346 | 7.65584E-10 | 37.84568472 |  |
| rs10896012 | 11 | 65278461 | C | T | 0.022403645 | 0.003007933 | 9.46314E-14 | 55.47515135 |  |
| rs10938398 | 4 | 45186139 | A | G | 0.028359316 | 0.002504427 | 1.00185E-29 | 128.2247796 |  |
| rs11030112 | 11 | 27705188 | A | G | 0.031328912 | 0.002647724 | 2.65461E-32 | 140.0046863 |  |
| rs113211479 | 11 | 47669861 | A | G | 0.024395151 | 0.00252038 | 3.6976E-22 | 93.68546249 | rs11039324(1.00) |
| rs7654647* | 4 | 80810453 | T | A | 0.015544534 | 0.002519704 | 6.864E-10 | 38.05869024 | rs11098930(1.00) |
| rs11119208 | 1 | 209211968 | A | G | 0.015068594 | 0.002541571 | 3.05064E-09 | 35.15104026 |  |
| rs11150745 | 17 | 78757626 | G | A | -0.019426656 | 0.002664949 | 3.10668E-13 | 53.13926037 |  |
| rs112154095 | 18 | 22204767 | T | C | -0.019877199 | 0.003184062 | 4.30034E-10 | 38.97127601 | rs111581974(1.00) |
| rs111610668 | 1 | 1548470 | G | A | -0.017000824 | 0.002557563 | 2.98569E-11 | 44.18598873 |  |
| rs113866544 | 17 | 46270606 | C | T | 0.036627543 | 0.004938617 | 1.20203E-13 | 55.00500539 |  |
| rs114593013 | 3 | 84113491 | G | A | -0.03690636 | 0.005324419 | 4.16318E-12 | 48.04578799 |  |
| rs11640322 | 16 | 9733274 | A | G | 0.016683014 | 0.002721626 | 8.79921E-10 | 37.57416207 |  |
| rs11679338 | 2 | 181606895 | C | T | -0.017247647 | 0.002614467 | 4.19552E-11 | 43.52018119 |  |
| rs11776713 | 8 | 30861607 | C | T | -0.015107302 | 0.002475172 | 1.03747E-09 | 37.25292517 |  |
| rs12072739 | 1 | 98315893 | G | A | 0.018090145 | 0.002969636 | 1.11715E-09 | 37.10863098 |  |
| rs1225060 | 3 | 131637173 | A | G | 0.023159726 | 0.002779248 | 7.87577E-17 | 69.43999705 |  |
| rs12459368 | 19 | 18459377 | G | A | -0.019853131 | 0.00279158 | 1.14547E-12 | 50.57725076 |  |
| rs12739999 | 1 | 32207990 | A | G | 0.021676646 | 0.003305477 | 5.46087E-11 | 43.00442943 |  |
| rs4648664 | 1 | 2584095 | C | A | -0.015424865 | 0.002492872 | 6.10915E-10 | 38.28601545 | rs12756665(0.99) |
| rs13017207 | 2 | 100841235 | A | G | -0.019643359 | 0.002534562 | 9.17491E-15 | 60.06520423 |  |
| rs13062093 | 3 | 35667057 | G | T | 0.019749243 | 0.002569193 | 1.50691E-14 | 59.08874396 |  |
| rs13097150 | 3 | 62433911 | T | C | 0.015351432 | 0.002560652 | 2.03327E-09 | 35.94132064 |  |
| rs13263674 | 8 | 14238841 | G | A | 0.01705551 | 0.002737489 | 4.65402E-10 | 38.81699008 |  |
| rs13331491 | 16 | 394343 | T | C | -0.017638825 | 0.00284811 | 5.89666E-10 | 38.35509779 |  |
| rs13337177 | 16 | 2175323 | T | G | -0.022823772 | 0.00324515 | 2.01856E-12 | 49.46553425 |  |
| rs13393304 | 2 | 637830 | A | G | -0.04447051 | 0.003277381 | 6.12213E-42 | 184.1143299 |  |
| rs1474518 | 8 | 116825690 | C | T | -0.01811734 | 0.002933986 | 6.6167E-10 | 38.13028338 |  |
| rs1591726 | 1 | 49963473 | T | C | 0.022329482 | 0.002654773 | 4.06286E-17 | 70.74566526 |  |
| rs1652376 | 18 | 21109466 | T | G | -0.020876764 | 0.002482933 | 4.16683E-17 | 70.6958173 |  |
| rs1724557 | 4 | 137094048 | C | A | 0.015576561 | 0.00252496 | 6.87085E-10 | 38.05674245 |  |
| rs1762509 | 1 | 107592959 | A | G | 0.015699713 | 0.002624339 | 2.19943E-09 | 35.7882739 |  |
| rs17770336 | 9 | 28414625 | T | C | 0.024723385 | 0.002640919 | 7.84928E-21 | 87.64015977 |  |
| rs17773430 | 18 | 57963117 | C | T | 0.01922109 | 0.002787632 | 5.38156E-12 | 47.54254974 |  |
| rs1784461 | 11 | 118937985 | A | G | 0.015683528 | 0.002522119 | 5.02269E-10 | 38.66818632 |  |
| rs1834144 | 18 | 40744790 | A | C | -0.018117555 | 0.002567779 | 1.71696E-12 | 49.78302652 |  |
| rs1861026 | 7 | 76605322 | T | C | 0.020214687 | 0.003326582 | 1.22671E-09 | 36.92621279 |  |
| rs1928496 | 13 | 31012904 | C | T | -0.021059876 | 0.00282664 | 9.29889E-14 | 55.50957109 |  |
| rs2020942 | 17 | 28546914 | T | C | 0.0151581 | 0.002536368 | 2.2826E-09 | 35.71595767 |  |
| rs2095484 | 9 | 11452408 | C | T | -0.015220876 | 0.002549362 | 2.36578E-09 | 35.64623801 |  |
| rs215628 | 7 | 32360096 | C | T | 0.015755115 | 0.00255008 | 6.48026E-10 | 38.17094284 |  |
| rs2172131 | 10 | 133978962 | T | C | 0.016870677 | 0.002511512 | 1.85072E-11 | 45.12237562 |  |
| rs217669 | 14 | 62360075 | C | T | 0.017486178 | 0.002783103 | 3.32147E-10 | 39.47557576 |  |
| rs2180454 | 14 | 29690513 | T | C | -0.019021263 | 0.002955584 | 1.22902E-10 | 41.41803894 |  |
| rs2229616 | 18 | 58039276 | T | C | -0.096027 | 0.008810006 | 1.15549E-27 | 118.8041583 |  |
| rs2239647 | 14 | 33292743 | A | C | 0.017439943 | 0.002492779 | 2.63023E-12 | 48.94632391 |  |
| rs3787075* | 20 | 25186502 | G | C | 0.017758134 | 0.002621904 | 1.26145E-11 | 45.87314634 | rs2268880(1.00) |
| rs2304608 | 5 | 87962298 | A | C | 0.029859527 | 0.00340771 | 1.9123E-18 | 76.77821773 |  |
| rs2307111 | 5 | 75003678 | C | T | -0.026013581 | 0.002535635 | 1.07597E-24 | 105.2504955 |  |
| rs245775 | 5 | 170532105 | A | G | -0.019589582 | 0.002789058 | 2.16028E-12 | 49.33241998 |  |
| rs2472297 | 15 | 75027880 | T | C | 0.016565676 | 0.002794738 | 3.07669E-09 | 35.13448501 |  |
| rs2477467 | 13 | 111971623 | T | C | 0.018092715 | 0.00291524 | 5.42634E-10 | 38.51731832 |  |
| rs2481665 | 1 | 62594677 | C | T | -0.017841763 | 0.002490353 | 7.81559E-13 | 51.32760909 |  |
| rs2499468 | 6 | 51809081 | C | A | -0.015628562 | 0.002604866 | 1.97614E-09 | 35.99685138 |  |
| rs254024 | 5 | 103944020 | T | G | 0.015586956 | 0.002495234 | 4.19242E-10 | 39.0208979 |  |
| rs2667761 | 15 | 77883130 | C | T | -0.015918151 | 0.002584083 | 7.27098E-10 | 37.94630829 |  |
| rs2820223 | 6 | 35009295 | C | T | 0.01506694 | 0.002520043 | 2.24728E-09 | 35.74633347 |  |
| rs28473022 | 14 | 101161855 | A | G | 0.034468123 | 0.005659774 | 1.12898E-09 | 37.08808584 |  |
| rs9985922* | 4 | 20099138 | C | G | 0.023862617 | 0.003717521 | 1.37208E-10 | 41.20279598 | rs28559775(1.00) |
| rs2859977 | 2 | 50240255 | T | C | 0.020429799 | 0.003353259 | 1.11147E-09 | 37.11856993 |  |
| rs2926614 | 8 | 76301610 | T | C | -0.022405317 | 0.003232399 | 4.16455E-12 | 48.04514154 |  |
| rs2926864 | 5 | 153213196 | A | G | 0.017481246 | 0.002623611 | 2.68206E-11 | 44.3959338 |  |
| rs2962082 | 16 | 62816628 | A | G | -0.015354665 | 0.002487558 | 6.71824E-10 | 38.10056961 |  |
| rs111768603 | 3 | 42329113 | T | G | -0.023621575 | 0.00393741 | 1.98209E-09 | 35.99099124 | rs34147849(1.00) |
| rs35060985 | 11 | 43693110 | A | G | 0.023354219 | 0.002674861 | 2.52427E-18 | 76.22991845 |  |
| rs35697587 | 14 | 47298505 | G | A | 0.016689583 | 0.002477266 | 1.61582E-11 | 45.38819742 |  |
| rs362307 | 4 | 3241845 | T | C | 0.028695542 | 0.004787044 | 2.04215E-09 | 35.93282421 |  |
| rs3737992 | 1 | 33234128 | A | G | -0.019821855 | 0.003310958 | 2.14085E-09 | 35.84086981 |  |
| rs3764002 | 12 | 108618630 | T | C | -0.016972736 | 0.002817017 | 1.69033E-09 | 36.30128737 |  |
| rs3784692 | 15 | 67988133 | C | T | -0.024385394 | 0.002530976 | 5.70148E-22 | 92.8283848 |  |
| rs3850986 | 15 | 73091283 | G | T | -0.016832523 | 0.002649815 | 2.12064E-10 | 40.35196945 |  |
| rs40067 | 5 | 107439012 | A | G | -0.024457105 | 0.003314171 | 1.58821E-13 | 54.45747651 |  |
| rs41286710 | 1 | 46383020 | T | C | -0.036901248 | 0.005712709 | 1.05049E-10 | 41.72488847 |  |
| rs4239060 | 17 | 1844519 | A | G | -0.025590789 | 0.003182911 | 8.9796E-16 | 64.64213328 |  |
| rs4402589 | 16 | 29954654 | T | G | -0.026272698 | 0.002494614 | 6.1664E-26 | 110.9174906 |  |
| rs4482463 | 2 | 205375909 | C | A | 0.03617955 | 0.004715118 | 1.67894E-14 | 58.87603108 |  |
| rs4500930 | 2 | 228985505 | T | C | 0.017080381 | 0.002607658 | 5.75041E-11 | 42.90335924 |  |
| rs4665896 | 2 | 26943117 | C | T | 0.01975691 | 0.002483649 | 1.79434E-15 | 63.27831171 |  |
| rs4743930 | 9 | 96445594 | T | C | 0.017068336 | 0.002868246 | 2.66849E-09 | 35.41170197 |  |
| rs4929923 | 11 | 8639200 | T | C | -0.017975762 | 0.002588218 | 3.7785E-12 | 48.23589315 |  |
| rs538656 | 18 | 57850422 | T | G | 0.045344859 | 0.002917506 | 1.79327E-54 | 241.5627108 |  |
| rs55726687 | 12 | 991306 | A | G | 0.02267325 | 0.00303593 | 8.1235E-14 | 55.77522004 |  |
| rs55742087 | 3 | 185830488 | T | C | -0.023318082 | 0.003207701 | 3.61071E-13 | 52.84394276 |  |
| rs56356382 | 19 | 4064057 | C | T | -0.021675528 | 0.003156004 | 6.5095E-12 | 47.16952929 |  |
| rs56398417 | 3 | 88024986 | T | C | -0.01651318 | 0.002684794 | 7.71743E-10 | 37.83003973 |  |
| rs577525 | 10 | 99769388 | T | C | -0.017668256 | 0.002498172 | 1.52195E-12 | 50.01958187 |  |
| rs583077 | 1 | 42421994 | G | T | 0.016293432 | 0.002488957 | 5.89841E-11 | 42.85364413 |  |
| rs591939 | 17 | 40698075 | G | A | 0.019038518 | 0.002861974 | 2.88678E-11 | 44.25193725 |  |
| rs59893724 | 5 | 80830788 | G | A | -0.018898978 | 0.00287987 | 5.29338E-11 | 43.06537456 |  |
| rs60886478 | 2 | 555978 | T | C | -0.028412897 | 0.004779439 | 2.76763E-09 | 35.3406515 |  |
| rs6096886 | 20 | 50951298 | G | A | -0.028390231 | 0.003156081 | 2.35358E-19 | 80.91685062 |  |
| rs61813293 | 1 | 156019075 | T | G | 0.023662033 | 0.003525763 | 1.93057E-11 | 45.03966138 |  |
| rs61903695 | 11 | 89922417 | G | A | 0.016831236 | 0.002838617 | 3.04082E-09 | 35.15732415 |  |
| rs61910767 | 11 | 134515899 | T | C | -0.022871978 | 0.00333471 | 6.94604E-12 | 47.04231069 |  |
| rs62007782 | 15 | 78029797 | A | G | -0.017409675 | 0.002805417 | 5.44415E-10 | 38.51091978 |  |
| rs62084234 | 17 | 65847060 | G | A | 0.025467412 | 0.003125296 | 3.67552E-16 | 66.40253898 |  |
| rs62106258 | 2 | 417167 | C | T | -0.076900387 | 0.005767158 | 1.46435E-40 | 177.7995472 |  |
| rs62107115 | 19 | 30289630 | A | G | 0.019282611 | 0.002649089 | 3.36404E-13 | 52.98292826 |  |
| rs62183012 | 2 | 159607760 | C | T | -0.016234134 | 0.002736075 | 2.96786E-09 | 35.20461371 |  |
| rs62190394 | 2 | 230624929 | T | C | 0.021935414 | 0.0026682 | 2.01723E-16 | 67.58530556 |  |
| rs62259475 | 3 | 82710898 | A | G | 0.01601685 | 0.002587954 | 6.05463E-10 | 38.30349622 |  |
| rs62261725 | 3 | 85898626 | G | A | -0.021447962 | 0.002648059 | 5.51846E-16 | 65.60152524 |  |
| rs6433243 | 2 | 171605495 | C | T | 0.016183174 | 0.002596073 | 4.55508E-10 | 38.85893453 |  |
| rs653958 | 1 | 96884006 | G | A | 0.018684232 | 0.002565698 | 3.28132E-13 | 53.03183351 |  |
| rs12335914 | 9 | 92209151 | C | G | 0.016019593 | 0.00248858 | 1.21664E-10 | 41.43782316 | rs6559365(0.91) |
| rs66679256 | 4 | 18351898 | T | C | 0.018498895 | 0.002497369 | 1.28844E-13 | 54.86855844 |  |
| rs113658831 | 1 | 210097016 | G | C | 0.023558931 | 0.003348078 | 1.97053E-12 | 49.51278427 | rs6695101(1.00) |
| rs6739755 | 2 | 59330227 | A | G | 0.022714138 | 0.002541235 | 3.95463E-19 | 79.89141064 |  |
| rs6788620 | 3 | 124691999 | A | G | 0.014944845 | 0.002515128 | 2.81598E-09 | 35.30691942 |  |
| rs68169458 | 8 | 67827207 | C | T | 0.016926716 | 0.002726873 | 5.38779E-10 | 38.5312291 |  |
| rs6870983 | 5 | 87697533 | T | C | -0.02250481 | 0.003076559 | 2.57516E-13 | 53.5078516 |  |
| rs704061 | 12 | 89771903 | C | T | 0.016451913 | 0.00249031 | 3.93851E-11 | 43.64388694 |  |
| rs7132908 | 12 | 50263148 | A | G | 0.025531303 | 0.002546892 | 1.18969E-23 | 100.4898314 |  |
| rs7156625 | 14 | 79942647 | A | G | 0.026865091 | 0.002980413 | 1.98886E-19 | 81.24959153 |  |
| rs7165759 | 15 | 80988600 | A | G | -0.017496348 | 0.002706758 | 1.02005E-10 | 41.78239268 |  |
| rs719802 | 11 | 113234679 | T | C | 0.017378811 | 0.00254021 | 7.83762E-12 | 46.80563563 |  |
| rs7243566 | 18 | 57707448 | T | C | -0.016896987 | 0.002853788 | 3.20189E-09 | 35.05681287 |  |
| rs112108364 | 13 | 86490590 | G | T | 0.017346135 | 0.002759729 | 3.26908E-10 | 39.50661958 | rs72632776(1.00) |
| rs72663503 | 1 | 39969059 | T | C | 0.020886958 | 0.00295812 | 1.65441E-12 | 49.85584189 |  |
| rs72995085 | 6 | 143193971 | C | T | -0.02147882 | 0.003248024 | 3.76894E-11 | 43.73001402 |  |
| rs7498665 | 16 | 28883241 | G | A | 0.026913788 | 0.002528198 | 1.8311E-26 | 113.3247691 |  |
| rs749953 | 12 | 108422857 | T | C | -0.017489299 | 0.002941149 | 2.74077E-09 | 35.3596492 |  |
| rs7550711 | 1 | 110082886 | T | C | 0.061532622 | 0.007866541 | 5.19625E-15 | 61.18437069 |  |
| rs76040172 | 21 | 46488959 | A | G | -0.0450907 | 0.005505258 | 2.60187E-16 | 67.08354274 |  |
| rs7608397 | 2 | 58769042 | T | G | -0.016853647 | 0.00251265 | 1.9796E-11 | 44.99054503 |  |
| rs76327888 | 14 | 103380403 | T | G | 0.022949627 | 0.003286813 | 2.90317E-12 | 48.75267999 |  |
| rs765123 | 12 | 2155997 | C | G | 0.019471457 | 0.003017842 | 1.10301E-10 | 41.62949998 |  |
| rs778094 | 2 | 147903802 | G | A | 0.014975339 | 0.002514027 | 2.5736E-09 | 35.48222254 |  |
| rs7822494 | 8 | 87476439 | C | T | -0.016236846 | 0.0024861 | 6.53101E-11 | 42.65435393 |  |
| rs7845090 | 8 | 73449940 | G | A | 0.020056041 | 0.002752647 | 3.1907E-13 | 53.08683605 |  |
| rs78719460 | 12 | 133395038 | A | G | 0.015897527 | 0.002675939 | 2.83435E-09 | 35.29425522 |  |
| rs7893571 | 10 | 16750129 | G | T | -0.017854819 | 0.002631778 | 1.16637E-11 | 46.02667052 |  |
| rs7942037 | 11 | 30430332 | C | G | -0.016889174 | 0.002577369 | 5.64402E-11 | 42.93989176 | rs7928173(1.00) |
| rs7982447 | 13 | 54453811 | C | T | 0.020753935 | 0.003080673 | 1.61893E-11 | 45.38442894 |  |
| rs8015400 | 14 | 25930988 | C | A | -0.019945799 | 0.002650134 | 5.2184E-14 | 56.6453816 |  |
| rs809955 | 4 | 140874760 | A | G | -0.015842556 | 0.002573445 | 7.45269E-10 | 37.89815096 |  |
| rs9320823 | 6 | 98429337 | T | C | -0.023204799 | 0.002531705 | 4.92356E-20 | 84.00917181 |  |
| rs9469899 | 6 | 34793124 | A | G | 0.023988724 | 0.002591778 | 2.1283E-20 | 85.66746424 |  |
| rs9471333 | 6 | 40362023 | C | T | 0.024393122 | 0.002487469 | 1.05668E-22 | 96.16495497 |  |
| rs9641499 | 7 | 112984493 | A | C | -0.01776751 | 0.002500591 | 1.20041E-12 | 50.48531073 |  |
| rs9832402 | 3 | 173742542 | G | A | -0.016955973 | 0.002858389 | 2.99262E-09 | 35.18843635 |  |
| rs9843340 | 3 | 156317073 | C | T | -0.022144713 | 0.00346929 | 1.73575E-10 | 40.74330879 |  |
| rs9867802 | 3 | 41343555 | C | A | 0.015530827 | 0.002617627 | 2.97138E-09 | 35.20230406 |  |
| rs9911991 | 17 | 42278916 | C | G | 0.019135455 | 0.002950357 | 8.82559E-11 | 42.06548089 | rs9910055(0.96) |
| rs9989141 | 14 | 94006257 | C | T | -0.020101054 | 0.002580824 | 6.77449E-15 | 60.66222891 |  |

snp: single nucleotide polymorphism; chr: chromosome number; pos: position of the lead SNP (GRCh37/hg19); a1: coding/effective allele; a2: the other allele; beta: estimate of SNP effect size; se: estimate of standard error; P: P-value, two-tailed and unadjusted (marginal z-test); proxy SNPs (r2>0.9 with the specified SNP in EUR population, searched in Phenoscanner, <http://www.phenoscanner.medschl.cam.ac.uk/> ) were used in the MEGASTROKE dataset.
